# Effect of Cooling and Annealing Conditions on the Microstructure, Mechanical and Superelastic Behavior of a Rotary Forged Ti–18Zr–15Nb (at. %) Bar Stock for Spinal Implants

**DOI:** 10.3390/jfb13040259

**Published:** 2022-11-21

**Authors:** Konstantin Lukashevich, Vadim Sheremetyev, Alexander Komissarov, Vladimir Cheverikin, Vladimir Andreev, Sergey Prokoshkin, Vladimir Brailovski

**Affiliations:** 1 Metal Forming Department, National University of Science and Technology MISIS, 119049 Moscow, Russia; 2 Laboratory of Hybrid Nanostructured Materials, National University of Science and Technology “MISiS”, 119049 Moscow, Russia; 3 Department of Physical Metallurgy of Non-Ferrous Metals, National University of Science and Technology “MISiS”, 119049 Moscow, Russia; 4 A. A. Baikov Institute of Metallurgy and Materials Science, Russian Academy of Sciences, 119334 Moscow, Russia; 5 Department of Mechanical Engineering, École de Technologie Supérieure, 1100 Notre-Dame Street West, Montreal, QC H3C 1K3, Canada

**Keywords:** Ti–Zr–Nb shape memory alloy, thermomechanical treatment, rotary forging, cooling rate, superelastic properties

## Abstract

In this work, the microstructure, phase state, texture, superelastic and mechanical properties of a Ti–18Zr–15Nb (at. %) shape memory alloy subjected to a combined thermomechanical treatment, including hot rotary forging with either air cooling or water quenching and post-deformation annealing are studied. It was revealed that the main structural component of the deformed and annealed alloy is BCC *β*-phase. With an increase in the forging temperature from 600 to 700 °C, the average grain size increases from 5.4 to 17.8 µm for the air-cooled specimens and from 3.4 to 14.7 µm for the water-quenched specimens. Annealing at 525 °C after forging at 700 °C with water quenching leads to the formation of a mixed statically and dynamically polygonized substructure of *β*-phase. In this state, the alloy demonstrates the best combination of functional properties in this study: a Young’s modulus of ~33 GPa, an ultimate tensile strength of ~600 MPa and a superelastic recovery strain of ~3.4%.

## 1. Introduction

Degenerative spine diseases are widespread in the general population. Over the past decade, the number of patients in need of treatment for these ailments has increased significantly, with the rate more than doubling among those younger than 20. In the case of degenerative changes in the lumbar spine, surgical interventions using spinal fixation systems (pedicle screws, rods, cages) are frequently needed. Recently, dynamic fixation systems allowing a certain mobility in the spinal motion segment have become widespread, thus reducing the number of complications stemming from the use of rigid fixation systems [1,2,3]. The dynamic correction of the spine using the vertebral body tethering technique is of particular importance for growing patients (children) [4]. To produce the spinal fixation rods used for these treatments, there is a clear need for biocompatible metallic low-stiffness 4–7 mm diameter bar stock [5,6,7].

Metallic biomaterials commonly used for orthopedic implants are mainly medical stainless steels, cobalt–chromium and titanium alloys [8,9,10,11]. Titanium and its alloys, such as Ti–6Al–4V and Ti–Ni shape memory alloys (SMAs), are widely used to manufacture biomedical implants due to their adequate biocompatibility, high corrosion resistance, and excellent ultimate strength [10,12,13,14,15,16]. However, the ion release of non-biocompatible elements (such as V, Al in Ti–6Al–4V alloy and Ni in Ti–Ni SMAs) may cause inflammation and allergic response or senile dementia of the Alzheimer type [17,18,19]. Another problem to consider is the mismatch between the stiffness of implants and bones, which may cause the “stress-shielding effect“ and adjacent segment degeneration [20,21]. 

In the last decade, new nickel-free metastable *β*-type titanium alloys containing non-toxic and highly biocompatible alloying elements have received increased attention and could be considered alternatives to both conventional Ti–6Al–4V and superelastic Ti–Ni alloys [22,23,24,25,26]. Recently, many quaternary Ti–Zr–Nb-X alloys with Sn, Ta and Fe as X additions have been designed [27,28,29,30,31,32,33,34]. For example, Ti–29Nb–13Ta–4.6Zr (wt. %) *β*–Ti alloy manifests a relatively low Young’s modulus (~55 GPa), a high ultimate tensile strength (~800 MPa) and a satisfactory elastic recovery strain of ~3% [35,36]. Animal experiments with rabbit [37] have shown that using less stiff Ti–29Nb–13Ta–4.6Zr bone plates delays or minimizes bone atrophy and promotes bone formation compared to their stiffer Ti–6Al–4V and 316L SS equivalents. Another study [38] comparing the stress-shielding effects caused by the use of Ti–24Nb–4Zr–8Sn (at. %) and Ti-6Al-4V plates for internal fixation in a porcine model led to a similar conclusion, that compliant Ti–Nb–Zr–Sn plates provide better biomechanical compatibility with surrounding tissues than their stiffer Ti64 equivalents. It must, however, be noted that these quaternary alloys are difficult to produce on an industrial scale because of large differences in the melting temperatures of the alloy components. In this context, ternary Ti–Zr–Nb alloys with an advantageous combination of strength and ductility, low Young’s modulus (<50 GPa) and superelastic behavior resulting from stress-induced reversible *β↔α*″ martensitic transformation with a maximum theoretical superelastic recovery strain as high as ~6% [39,40,41,42] appear to be less expensive, but promising, alternative implant materials.

To improve the mechanical and functional properties of *β*-type SMAs, such as Ti–Zr–Nb, their microstructure, phase composition and texture can be controlled by thermomechanical treatments (TMTs), which typically include cold rolling (CR) and post-deformation annealing (PDA) [23,24,25,27,29,30,32,33,34,40,41,42,43]. In most of the mentioned works, severe CR with high accumulated true strains (*e* ≥ 3.0) was applied [23,24,25,27,29,30,32,34,40] and PDA was carried out in vacuum furnaces. Moreover, in these works, TMT was realized under laboratory conditions using small (thin foil) samples. Implementing severe cold deformation and vacuum heat-treatment operations in the production line for long-length bar stock still represents a significant technological challenge.

The combination of radial shear rolling, rotary forging and post-deformation annealing operations constitutes a promising TMT workflow for obtaining high-quality long-length SMAs bar stock [44,45,46,47]. Radial shear rolling (RSR) is a technologically effective method of producing semi-finished products, but the smallest diameter of round bars obtained by this method is 10 mm, and the structure formed over the bar cross sections is strongly heterogeneous [46,47]. To obtain the required 3 to 8 mm-diameter bars with high surface quality and homogeneous grain structure, the RSR operation can be followed by the rotary forging (RF) operation [44,47]. For additional control of the structural and phase state, the deformed material can also be submitted to post-deformation annealing [45]. Considering that, in some cases, the RF–PDA combination cannot entirely eliminate the heterogeneity resulting from the RSR operation, the latter can also be replaced by multi-axial forging [48].

It was shown in [49] that both the low-temperature TMT (LTMT), including cold RF and PDA, and the high-temperature TMT (HTMT), consisting of RF at 600–700 °C, could serve for the production of Ti–Zr–Nb long-length bar stock. Note that LTMT and HTMT contribute to the formation of different structural and phase states, and as a consequence, different sets of static and fatigue mechanical properties. For example, LTMT forms a mixed microstructure composed of the statically recrystallized structure and the polygonized dislocation substructure, which provides excellent static mechanical properties: a relatively high ultimate tensile strength (*UTS* = 680 MPa), a low Young’s modulus (*E* < 40 GPa) and a moderately high superelastic recovery strain (*ε_r_^se^_max_* = 3.4%). On the other hand, HTMT forms a dynamically polygonized substructure of *β*-phase with a uniform crystallographic texture close to the <101> direction, which corresponds to a maximum value of the theoretical recovery strain (*ε_β↔α_^max^* = 5.7%). The alloy in this state exhibits a stable functional tensile cyclic behavior and a superior fatigue resistance but a lower mechanical strength (*UTS =* 590 MPa).

It must be noted that, after all the TMT sequences, the alloy must be cooled down and the cooling rates may differ significantly, such as for the Ti–Nb alloy in [50], wherein the experimentally measured cooling rates in the 700 to 200 °C temperature range varied from 4–1 °C/s for air cooling (AC) to 350–100 °C/s for water quenching (WQ). In [51,52], the effect of the cooling rate on the structure, texture and functional properties of Ti–24Nb–4Zr–8Sn (wt. %) alloy was investigated. It was found that, with an increase in the cooling rate from AC to WQ, the average grain size increased from 68 to 81 µm without affecting the crystallographic texture. However, the WQ samples did not demonstrate superelasticity because the martensitic transformation temperatures after this treatment were too high. On the contrary, the AC samples manifested a superelastic behavior, but their superelasticity was limited: at stresses higher than 400 MPa, superelastic strains decreased while residual strains increased significantly. 

Furthermore in [53], the hot-deformed Ti–20.6Nb–13.6Zr–0.5V (wt. %) alloy was solution treated at 850 °C (above *β* transus temperature) and then either water-quenched or air-cooled. It was demonstrated that, with an increase in the cooling rate, the amount of *α″*-martensite within the *β* grains increased. After WQ, the samples manifested a lower Young’s modulus and a higher elongation to failure (59 GPa and 21%, respectively) compared to their AC counterparts (75 GPa and 13%, respectively). It was also revealed that the AC samples showed a greater hardness (240 HV), ultimate tensile strength (696 MPa) and yield stress (570 MPa) compared to the WQ samples (220 HV, 670 MPa and 516 MPa, respectively). The results of the structural studies and mechanical tests correlated well with those in [54], wherein the Young’s modulus of the Ti–20Nb (wt. %) alloy decreased with an increase in the cooling rate.

A thorough analysis of the body of works on Ti–Zr–Nb-based alloys has shown that the impacts of the cooling rate on the microstructure and mechanical behavior of these alloys have yet to be studied. To start filling this information gap, it is hypothesized that replacing AC by WQ at the last stage of TMT would allow freezing the structural and phase states of the alloy formed as a result of HTMT and prevent the development of softening processes. Moreover, it is also proposed to modify the initial step of a TMT sequence, by replacing rotary shear rolling with multi-axial forging with the objective of reducing the microstructure heterogeneity and increase the process productivity. To evaluate the impact of both modifications, in this work, Ti–18Zr–15Nb alloy samples were subjected to low- and high-temperature TMT with different cooling rates and annealing conditions, and their structural and phase states and their mechanical and superelastic properties were studied.

## 2. Materials and Methods

### 2.1. Materials Production and Treatment

A 20 kg-weight, 160 mm-diameter ingot with a nominal atomic composition Ti–18Zr–15Nb (at. %) was fabricated by vacuum arc melting. The ingot was re-melted at least 3 times to ensure compositional homogeneity. The chemical composition of the alloy was determined by energy-dispersive X-ray spectrometer point analysis using a field-emission scanning electron microscope (“JSM-7610F”, JEOL Ltd., Tokyo, Japan), and the obtained results are provided in Table 1. Additionally, concentrations of the impurities (O, N, H) were measured using a gas analyzer (“TC 600”, “CS 600”, “RHEN-602”, Leco, Geleen, The Netherlands), and the results are also collected in Table 1.

**Figure 1:**
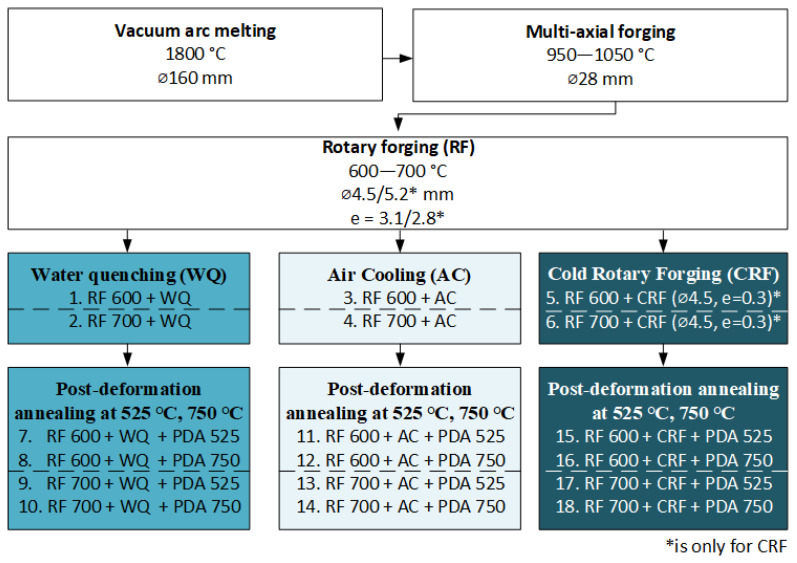
Technological TMT schedule.

The obtained ingot was subjected to multi-axial hot forging in the 950–1050 °C temperature range with a true strain of e = 3.5 and then machined and ground to a 21.5 mm-diameter bar stock to remove the oxide layer and clean off surface defects. Then the bars were rotary forged (RF, e = 3.1) at 600 °C and 700 °C with either AC or WQ to obtain 4.5 mm-diameter bar specimens. Two bars formed by RF at 600 °C and 700 °C with AC were additionally cold-rotary-forged (CRF, e = 0.3) to produce ~4.0 mm-diameter bar specimens having a microstructure similar to the one formed by a conventional low-temperature TMT [44,49]. The bars manufacture was realized in the industrial conditions of a production workshop (Matek-Sma Ltd., Moscow, Russia) in air under atmospheric pressure. Finally, all of the bars, RF and CRF, were subjected to PDA at 525 °C and 750 °C for 30 min under argon atmosphere and then WQ-ed. A PDA temperature of 525 °C was chosen for the formation of a statically polygonized *β*-phase substructure according to [44]. To obtain a fully recrystallized reference microstructure, PDA at 750 °C was carried out. In that way, 18 different types of specimens were obtained. The entire TMT schedule of this study is summarized in Figure 1.

### 2.2. Experimental Procedure

Three mm-thick cylindrical disks and 15 mm-long semi-cylindrical specimens were cut from the TMT-processed bars for the microstructure investigations of the transversal and longitudinal cross sections of bar specimens. The surfaces of interest were first ground on emery paper with a grain size ranging from 320 to 4000. Next, polishing was carried out using a “SAPHIR 560” (ATM GmbH, Germany) polishing machine for 20 min under 30 N of load with an “Eposil F” (ATM GmbH, Germany) suspension containing 0.1 μm SiO_2_ particles. During polishing, ammonia, hydrogen peroxide 3% and liquid soap were added to the suspension. After polishing, the specimens were cleaned in an ultrasonic bath with isopropyl alcohol for 10 min. To reveal the grain boundaries, surface etching was performed in a 1HF:3HNO_3_:6H_2_O solution for 20–40 s. The grain microstructure was analyzed using a “Versamet-2” (Union, Tokyo, Japan) optical microscope equipped with a “Nikon D90” (Nikon Corp., Tokyo, Japan) camera. The average grain size of *β*-phase was determined using the Heyn lineal intercept procedure [55].

Phase identification was realized on the previously prepared specimens using a “X’pert Pro” (Malvern Panalytical, Almelo, Netherlands) X-ray diffractometer in the 30°–90° 2θ range (*Cu_Kα_* radiation). The severity of lattice defects was assessed by measuring, for different material states, the half-width (*B_hkl_*) of the *β*-phase diffraction lines. Beta-phase lattice parameters (LP) were calculated from the angular coordinates of the centers of gravity of the *β*-phase XRD line profiles. The theoretical limit of recovery strain ($\varepsilon_{\beta ↔\alpha }^{max}$) corresponding to a maximum transformation lattice strain was calculated using LPs of the parent *β* (*a_β_*) and martensite *α″* (*b_α″_*) phases determined from the (110)*_β_* and (020)*_α″_* XRD peak angular coordinates [56]:

(1) $$\varepsilon_{\beta ↔\alpha }^{max}\;=\;\frac{b_{\alpha^{\prime \prime }}-\sqrt{2}a_{\beta }}{\sqrt{2}a_{\beta }}\;\;$$

To analyze the microstructure after various TMTs, electron back-scattered diffraction (EBSD) analysis was conducted using a “TESCAN VEGA LMH” (Tescan s.r.o., Brno, Check Republic) scanning electron microscope equipped with an electron backscatter diffraction unit was used. The texture evolution was characterized using the Aztec v3.1 EBSD software (Oxford Instruments, Abingdon, UK).

**Figure 2:**
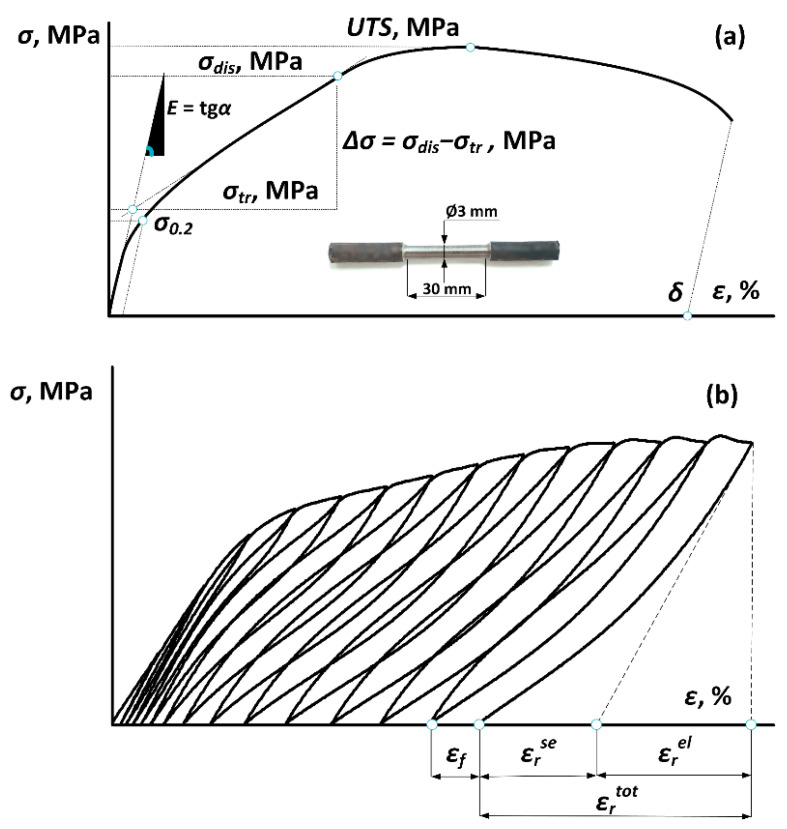
Metrics derived from the tensile stress–strain diagrams for (**a**) static and (**b**) cyclic tests.

To characterize the mechanical and functional properties, tensile static and cyclic tests were carried out at room temperature with a strain rate of 2 mm/min using an “Instron 5966” (Instron, Norwood, MA, USA) universal testing machine. The specimen gage diameter and length were, respectively, 3 and 30 mm (Figure 2a). From monotonic “stress–strain” curves, the Young’s modulus *E*, the relative elongation to failure *δ*, the transformation yield stress *σ_tr_*, the dislocation yield stress *σ_dis_*, the difference between the dislocation and transformation yield stresses *Δσ* = *σ_dis_ − σ_tr_* and the ultimate tensile strength *UTS* were determined as shown in Figure 2a. If no “double yielding” phenomenon appeared in the stress–strain diagram, the apparent yield stress *σ_0,2_* was calculated. All the measurements were carried out using at least three specimens, the results were averaged, and the confidence errors were calculated for each experimental point. Next, superelastic loading–unloading cyclic testing to failure was carried out on the same bone-shaped specimens by incrementally increasing the strain by 1% in each subsequent cycle. Finally, the elastic recovery strain *ε_r_^el^*, the superelastic recovery strain *ε_r_^se^*, the residual strain *ε_f_* and the total (elastic + superelastic) recovery strain *ε_r_^tot^* were measured as shown in Figure 2b.

## 3. Results

### 3.1. Influence of TMT on the Microstructure, Phase Composition and Texture

**Figure 3:**
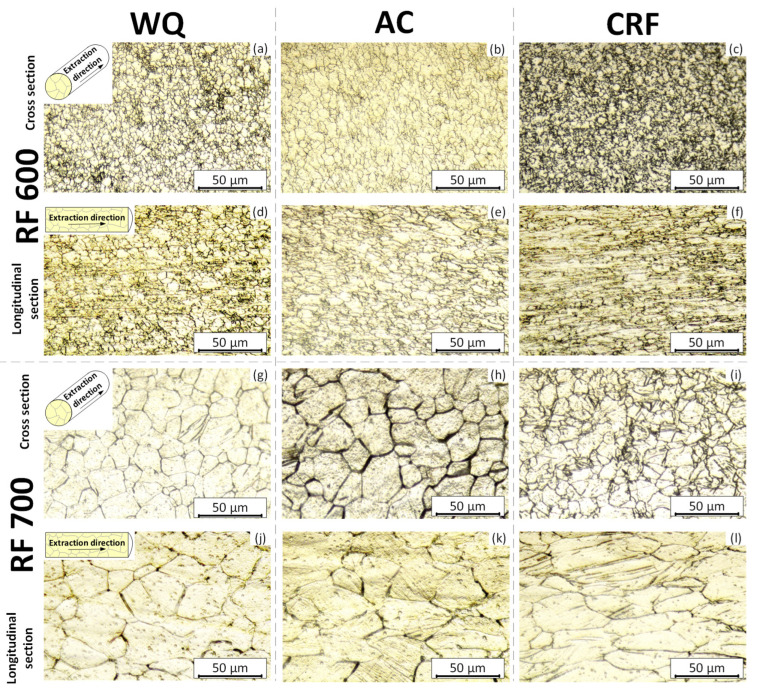
Optical micrographs of the Ti–18Zr–15Nb alloy after: (**a**–**f**) RF 600; (**g**–**l**) RF 700 with different final treatments.

**Figure 4:**
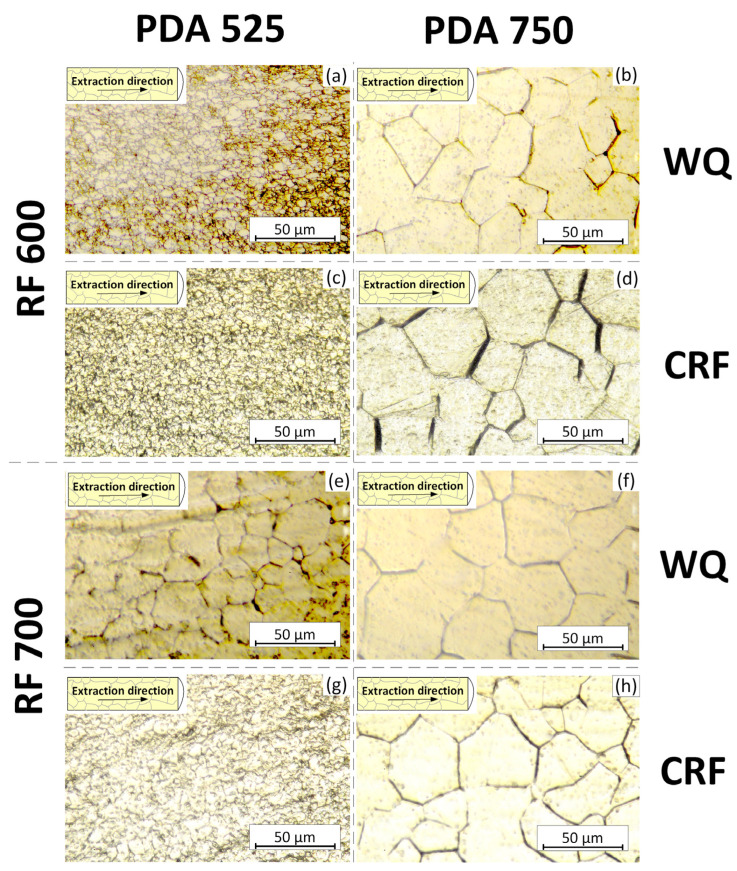
Optical micrographs of the Ti–18Zr–15Nb alloy after PDA at: (**a**,**c**,**e**,**g**) 525 °C; (**b**,**d**,**f**,**h**) 750 °C.

**Figure 5:**
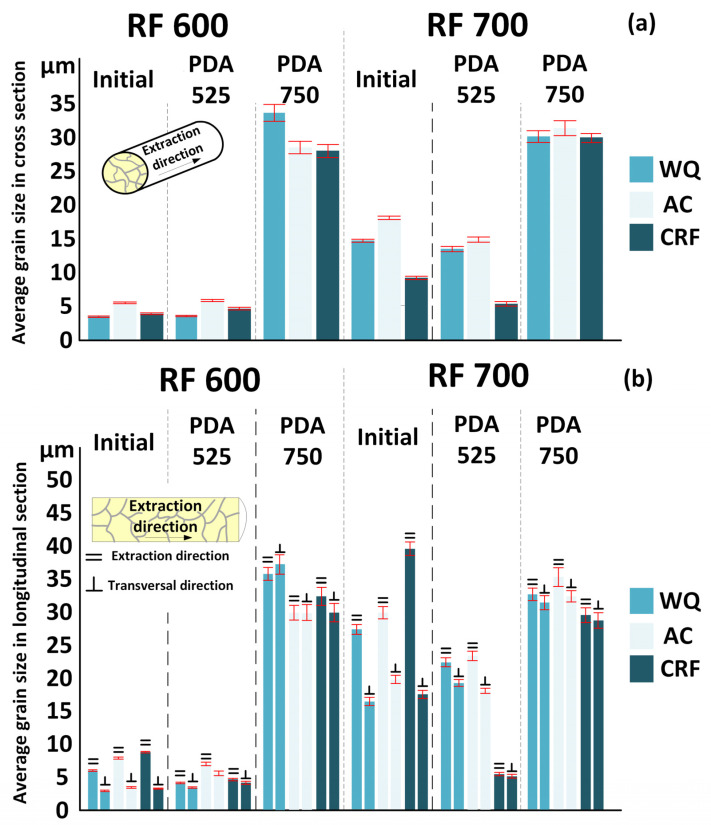
Average grain size of the *β*-phase: (**a**) transversal cross section; (**b**) longitudinal cross section.

Figure 3 and Figure 4 show optical micrographs of the microstructures observed in the transversal and longitudinal specimen cross sections. Histograms of the average grain size in both cross sections are presented in Figure 5. It can be observed that RF at 600 and 700 °C led to the formation of uniform cross-sectional microstructures with grains having an average size of 3–5 and 10–20 μm in the transversal and longitudinal cross sections, respectively. It is worth noting that the average grain size in the AC specimens is systematically higher than in their WQ equivalents because lower cooling rates facilitate grain border migration. It can be seen from Figure 3d–f,j–l, that RF, regardless of the temperature and cooling rate, leads to the formation of elongated grain microstructures: the grain size measured along the ED (extraction direction) is noticeably greater than that measured in the transversal direction (⊥ED). The most elongated grains form as a result of CRF, with an average grain size of (ED × ⊥ED) = 8.7 × 3.2 µm after RF at 600 °C and 40 × 18 µm after RF at 700 °C.

PDA at 525 °C affects the grain microstructure differently, depending on the RF conditions (RF600 and RF700) and the cooling rate applied (WQ and CRF), (Figure 4a,c,e,g). After RF700 + CRF + 525 °C, due to the static recrystallization process, the grain microstructure becomes finer, with an average grain size ≤10 μm (Figure 4g). After RF600 + CRF + 525 °C, grains become equiaxed, but their average size remains at the same level of ~5 μm (Figure 4c). PDA at 525 °C after either WQ or AC changes the shape of grains to a more equiaxed one as compared to the prior-to-PDA state, without modifying the average grain size (Figure 4a,e and Figure 5a). As expected, after PDA at 750 °C, a statically recrystallized microstructure with grains having an average size of 29–35 μm, regardless of the forging and cooling conditions, is formed (Figure 4b,d,f,h and Figure 5).

**Figure 6:**
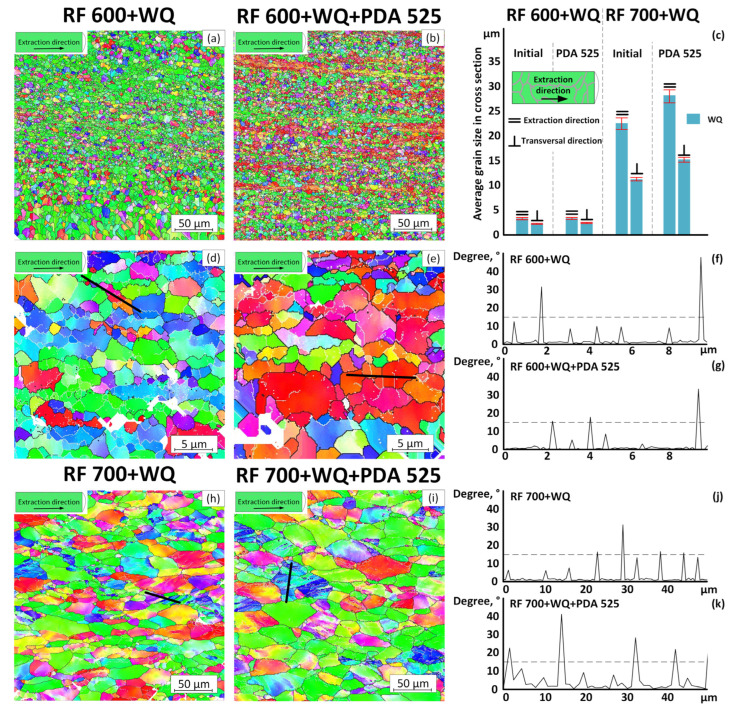
EBSD images of WQ specimens: (**a**,**d**) RF 600; (**b**,**e**) RF 600 + PDA 525; (**h**) RF 700; (**i**) RF 700 + PDA 525. Black lines correspond to the high-angle boundaries (>15°), and white lines to the low-angle boundaries (subboundaries) (3–15°); (**f**,**g**,**j**,**k**) misorientation profile scans in some selected areas marked with a bold black line; (**c**) average grain size of *β*-phase measured in the longitudinal cross section using the EBSD images.

For a more detailed analysis of the microstructure evolution, as well as to clarify features related to the recrystallization or grain-boundaries migration, the EBSD images, the histogram of an average grain size and the scans of misorientation profiles of the RF (600 and 700 °C) + WQ + PDA (525 °C) specimens are presented in Figure 6. These TMT conditions were selected for more detailed analysis because they had not been studied before. It can be seen in Figure 6a,d,f that RF at 600 °C leads to the formation of a fine-grained microstructure (average grain size of ~4 μm) composed of the recrystallized equiaxed grains and grains stretched in the extraction direction and containing a polygonized dislocation substructure (subgrains separated by low-angle boundaries). After PDA at 525 °C, the grain microstructure remains practically unchanged (Figure 6b,e): some equiaxed and elongated grains are retained, while the grain size increases slightly due to the high-angle-boundaries migration. After RF at 700 °C (Figure 6h), the grain microstructure is composed of strongly elongated grains with a clearly visible substructure. The subgrain boundaries in this case were presumably formed during the redistribution of dislocations into energetically more favorable walls. Some areas consist of structural elements separated by parallel low-angle boundaries (misorientation angle 10–14°) and high-angle boundaries (misorientation angle 15–17°) (see Figure 6h,j). These observations are in line with [49], and they reflect a continuous increase in the subgrain misorientations caused by the subgrain coalescence, and thus, represent an initial stage of the continuous dynamic recrystallization [57]. After PDA at 525 °C, the number of such mixed areas decreases significantly, and the misorientation angles at the preserved parallel boundaries increase to reach 20–40° (Figure 6i,j). The size of non-equiaxed grains increased slightly in all directions due to the high-angle-boundaries migration (Figure 6c,i). Thus, PDA at 525 °C after RF at 700 °C leads to the development of the recrystallization and polygonization processes, which are witnessed by an increase in the subgrain/grain misorientations and the migration of high-angle boundaries.

**Figure 7:**
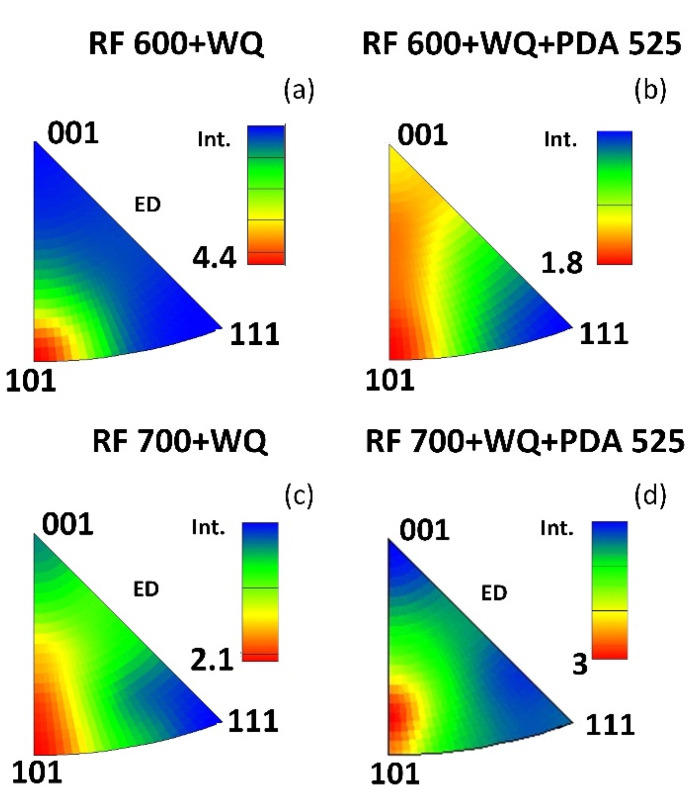
Inverse pole figures for specimens with WQ after: (**a**) RF 600; (**b**) RF 600 + PDA 525; (**c**) RF 700; (**d**) RF 700 + PDA 525.

The main functional properties of biomedically metastable *β*-Ti SMA, such as recovery strains and Young’s moduli, are highly sensitive to the crystallographic texture [40,46,58,59]. For Ti–18Zr–14Nb SMA, the <101> orientation corresponds to a maximum theoretical recovery strain of ∼6%, and conversely, the <001> and <111> orientations correspond to relatively low theoretical recovery strains of ∼2% [46]. Furthermore, it was experimentally measured for Ti–Nb–Zr–Sn that the <001> and <111> orientations correspond, respectively, to the minimum and maximum Young’s moduli (26 and 87 GPa) [58]. For the alloy of this study, the inverse pole figures with intensity scales calculated from the EBSD images of Figure 6a,b,h,i are shown in Figure 7. It can be seen that for specimens after RF at 600 °C, a strong crystallographic texture in the <101> direction is formed (Figure 7a). As a result of PDA at 525 °C, the maximum of texture intensity morphs towards the <001> direction (Figure 7b), which must contribute to a decrease in both the Young’s modulus and the recovery strain. According to [39], in this direction, the theoretical limit of the recovery strain can be estimated as ~4%. The texture after RF at 700 °C is similar to that after RF (600 °C)-PDA (525 °C) but is more pronounced in the <101> direction, which is advantageous from a recovery strain viewpoint. In general, for the considered specimens, a favorable texture forms after TMT of this alloy, and this texture corresponds to a 4–6% range of the theoretical recovery strain.

**Figure 8:**
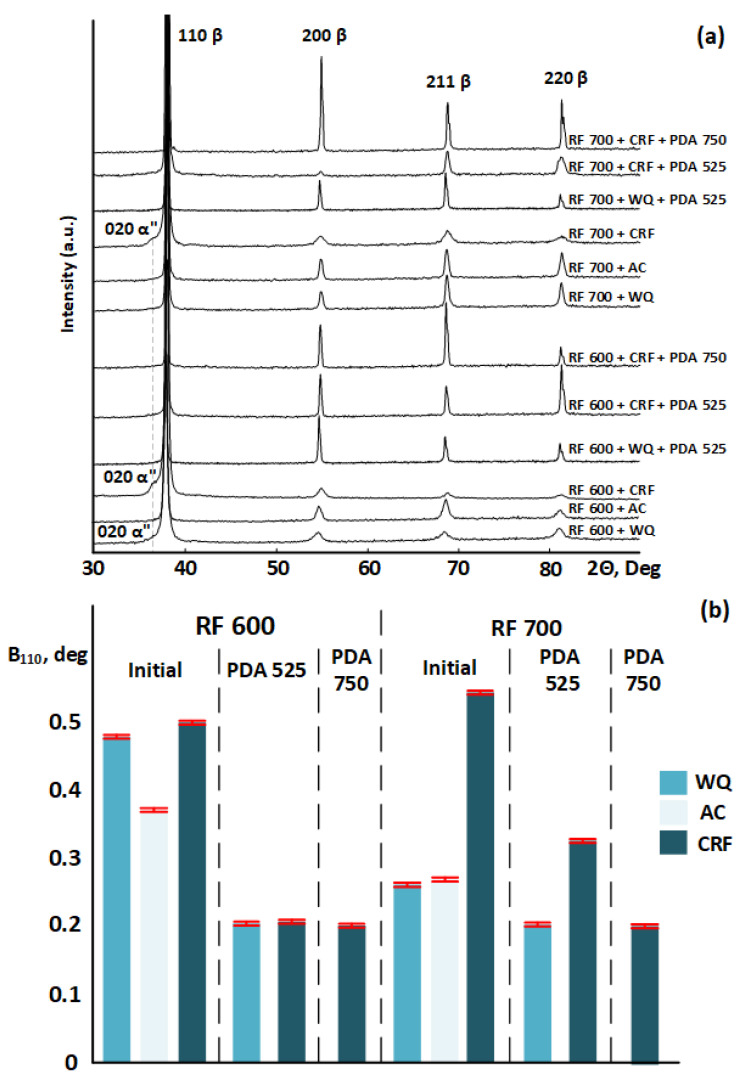
(**a**) X-ray diffraction profiles of the Ti–18Zr–15Nb alloy after various TMTs; (**b**) the width of the *β*-phase X-ray line of the Ti–18Zr–15Nb alloy after various TMTs.

Figure 8a shows the XRD patterns after different TMTs, wherein the main phase constituent is BCC *β*-phase. CRF leads to the formation of a low amount of stress-induced and stabilized *α*″-martensite. Changes in the cooling conditions do not lead to the precipitation of secondary *α-* or *ω*-phases. Variations in the *β*-phase X-ray line width shown in Figure 8b reflect the evolution of lattice defectness, as a result of the applied TMT routes. After RF at 600 °C, the X-ray B_110_ line width is greater than after RF at 700 °C, which indicates a more significant substructural hardening of the material in the former case. No observable differences in the B_110_ line width were found between the WQ and AC conditions, which suggests that the substructural hardening is not significantly affected by the cooling rate variations. A decrease in the line half-width after PDA points to polygonization- and recrystallization-induced material softening and correlates with the optical microscopy results. Note that the wider X-ray B_110_ line observed after RF 700 °C and PDA at 525 °C may indicate an incomplete recrystallization. After all the TMTs, the corresponding lattice parameter of *β*-phase (BCC) is estimated to be *a* = 3.344 ± 0.004 Ǻ, which is close to the values found for the Ti–18Zr–15Nb alloy in [40,41]. For the alloy of this study subjected to RF600 + CRF, the maximum calculated theoretical recovery strain is *ε_β↔α_^max^* = 4.8 ± 0.8%, wherein a large estimation error is related to the strong (020)*_α″_* X-ray line broadening.

### 3.2. Influence of TMT on the Mechanical and Superelastic Properties

**Figure 9:**
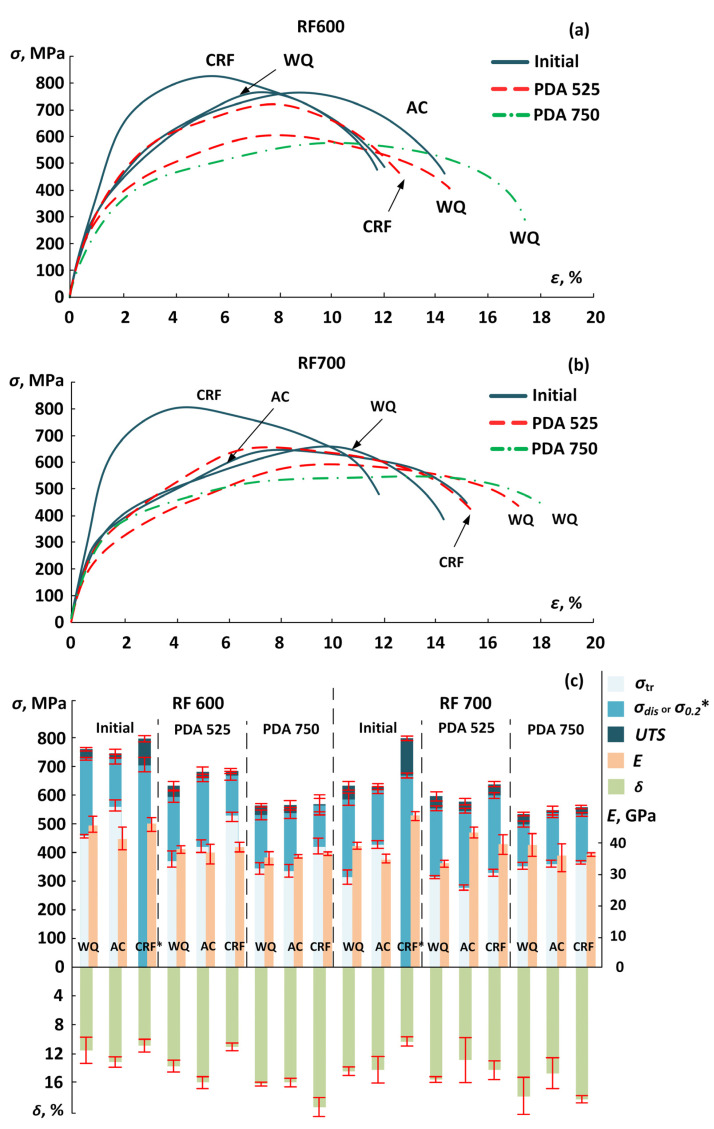
(**a**,**b**) Tensile stress–strain curves and (**c**) mechanical properties of the Ti–18Zr–15Nb alloy after TMT. *—*σ*_0,2_ was calculated for (RF600/RF700) + CRF.

Static tensile “stress–strain“ engineering diagrams for the most indicative TMTs are shown in Figure 9a,b, and the corresponding mechanical properties are presented in Figure 9c. After TMT, the alloy demonstrates a relatively low apparent Young’s modulus (33–51 GPa). After RF at 600 °C, it exhibits a relatively high strength (*UTS* = 750–760 MPa) regardless of the cooling conditions, compared to only ~630 MPa after RF at 700 °C, which is due to the combined effects of the fine-grained microstructure and the high dislocation density. CRF after RF600 leads to a slight increase in the *UTS* value (from 747 to 800 MPa), which contrasts with RF at 700 °C, wherein such hardening is much greater (from 632 to 801 MPa). After PDA at 525 °C, the ultimate tensile strength is reduced to 635–685 MPa and depends on the cooling conditions. After PDA at 750 °C, the specimens are characterized by a lower strength of 540–570 MPa because of the coarser microstructure and the lower level of dislocation density. It is noteworthy that, after reaching the ultimate tensile strength, the stress–strain diagrams show a steady decline in stress, which is associated with necking. A “double yielding” phenomenon visible on the stress–strain diagram indicates the development of stress-induced martensitic transformation. This is typical for all of the specimens, except for the CRF specimens before annealing, as shown in Figure 9a,b.

Note that the transformation and dislocation yield stresses are systematically higher for the AC specimens as compared to their WQ equivalents. Thus, after RF at 600 °C and WQ, the transformation yield stress is ~460 MPa, while after AC, it increases to ~560 MPa. Like the ultimate tensile strength, the transformation and dislocation yield stresses decrease after PDA. Normally, the greater the difference between the dislocation and transformation yield stresses, the higher the superelastic strain recovered upon unloading. However, in this work, no correlation was seen between these parameters because of large direct measurement errors. After different TMTs, the elongation to failure is greater than 10%. After the PDA, the ductility increases, regardless of the RF temperature and the cooling conditions, and the maximum 15–20% elongation to failure is seen in specimens annealed at 750 °C.

**Figure 10:**
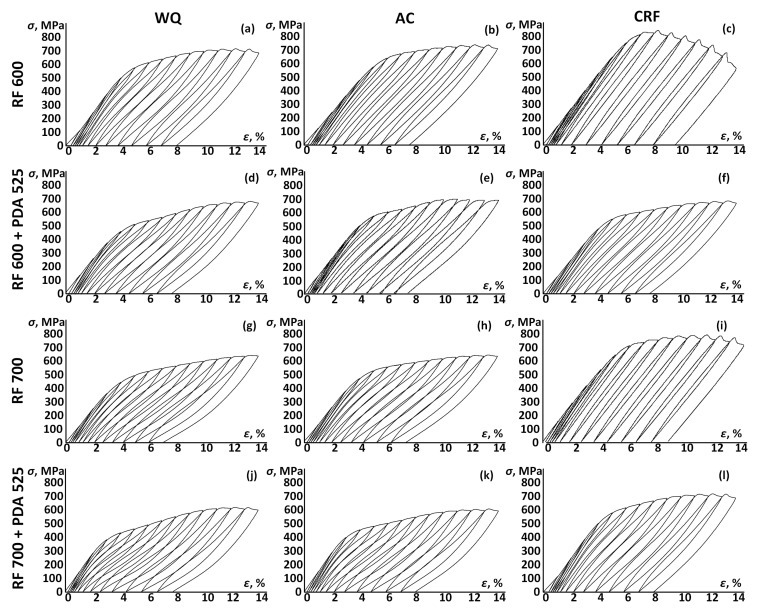
Stress–strain curves obtained during cyclic tensile testing: (**a**–**c**) RF 600; (**d**–**f**) RF 600 + PDA 525; (**g**–**i**) RF 700; (**j**–**l**) RF 700 + PDA 525.

**Figure 11:**
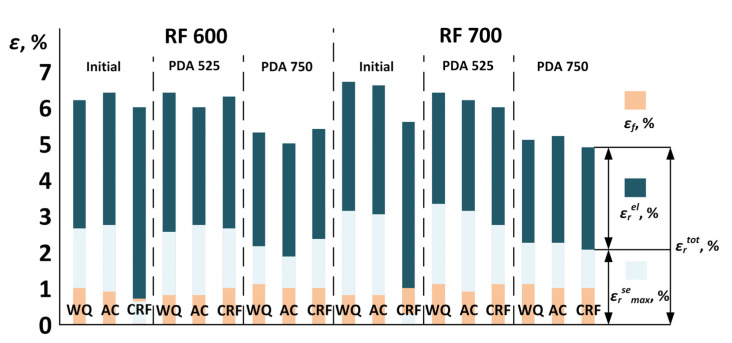
Elastic, superelastic and total recovery strains of the Ti–18Zr–15Nb alloy after TMT.

Figure 10 shows the cyclic loading–unloading tensile curves from 0 up to 14% of the applied strain after different TMT conditions. Values of the total recovery and residual strains, as well as the maximum superelastic strain recovered in a cycle due to reverse martensitic transformation, are plotted in Figure 11.

Note that, after CRF, the alloy does not manifest significant superelasticity (ε_r_^se^ < 1%). In this state, the material deforms mainly by the elastic and, probably, partially martensitic transformation mechanisms without accumulating significant irreversible strains. For all other specimens, the superelastic effect is clearly observable. A sloped stress plateau is also visible on both the loading and unloading curves, which is associated with the mechanical (stress) hysteresis due to the reversible *β↔α″* transformation. For all the specimens, except for CRF, the ε_r_^el^ and ε_r_^se^ values increase up to a maximum value at 10–13% strain and then decrease. The maximum ε_r_^se^ values after RF700 + WQ + PDA525 amount to ~3.4%. Specimens after RF at 700 °C demonstrate higher superelastic recovery strains as compared to their equivalents after RF at 600 °C. This can be due to a sharper <101> texture in the former case as compared to the latter. Without PDA, the superelastic recovery strain of the alloy after RF at 700 °C is 3.1–3.2%. After PDA at 525 °C, the superelastic recovery strain increases by 0.1–0.2%. PDA at 525 °C after RF at 600 °C does not affect the superelasticity. It is also worth noting that the superelastic recovery strain was not found to be dependent on the cooling rate. After PDA at 525 °C, the total recovery strain varies between 6–6.7% for the WQ and AC specimens. After PDA at 750 °C, the total and superelastic recovery strain values decrease.

Thus, it can be concluded that an increase in the RF temperature to 700 °C has a positive effect on the functional characteristics of the alloy and PDA at 525 °C maximizes the alloy’s superelastic characteristics. This improvement in properties is related to the formation of a polygonized substructure of *β*-phase during high-temperature deformation and to the formation of a preferable crystallographic texture with the maximum intensity in the <101> direction, during post-deformation annealing.

## 4. Discussion

The present study revealed that cooling rate does not play a significant role in the microstructure formation, since no large differences in the mechanical behavior of the alloy subjected to different cooling rates were found. It was expected that rapid cooling after RF would preserve the dynamically formed microstructure and prevent the development of static recrystallization. In the course of the work, it became clear that longer cooling times in air after RF at 600 °C and 700 °C were not enough for the radical development of primary recrystallization, contrary to what was shown in [44,47]. However, for samples with AC, a slight increase in the average grain size was observed. At the same time, after RF600 + AC, the stress relaxation phenomenon associated with a decrease in the density of lattice defects led to a decrease in the transformation yield stresses, thus facilitating the realization of direct martensitic transformation during loading. A phase composition analysis showed that WQ did not lead to the precipitation of secondary α-, α”- or ω-phases, contrary to what was shown in [52,53,54], which could have strongly affected the mechanical properties of the alloy of this study.

The PDA temperature of 525 °C after RF at 600 ° and 700 °C was not enough to induce the emergence of centers of primary recrystallization. At this temperature, non-equiaxed grains somewhat increased in size, as a consequence of the grain-boundary migration to more favorable energy positions. At the same time, the material softened without significant changes in its superelastic properties because of the polygonization processes, which was confirmed by the X-ray analysis, EBSD and mechanical tests. As expected, after PDA at 750 °C, a recrystallized microstructure with an equivalent average grain size of ~30 μm was formed in all the samples, regardless of the pre-treatment. In this case, the ductility of the alloy increased, but at the expense of lower strength and functional characteristics.

In previous works [44,46,47], a combination of the RSR + RF operations was effectively used to produce long-length bar stock. However, after RSR, a significantly heterogeneous microstructure was formed over the transversal cross section of the bar stock. In this paper, a decision was made to replace RSR with hot multi-axial forging. As a result of RF at 600° and 700 °C applied to the forged alloy, a more homogenous grain microstructure with a stronger <101> crystallographic texture was obtained, as compared to the RSR + RF case. This replacement positively affected the functional properties of the alloy: after RF at 600 °C, the superelastic strain in this work was greater than in [44]: 2.8 versus 2.3%. It can therefore be stated that the main parameter affecting the microstructure, mechanical and functional characteristics in this study was the RF temperature. An increase in the RF temperature significantly increased the initial grain size, which reduced the strength characteristics of the material, while slightly increasing the superelastic recovery strain.

**Figure 12:**
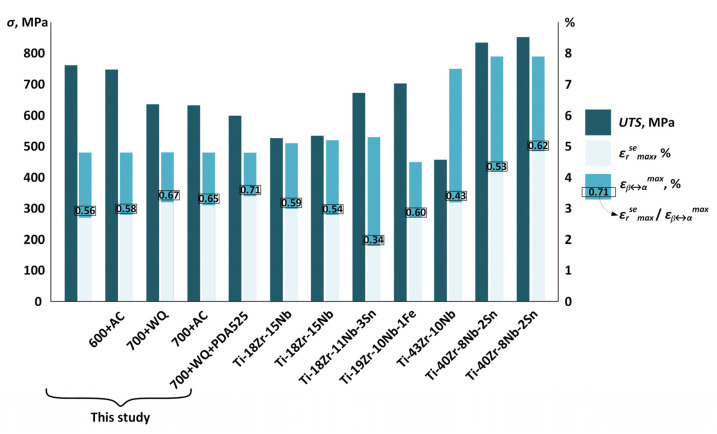
Comparison of the strength (*UTS*) and superelasticity (*ε_r_^se^_max_, ε_β↔α_^max^, ε_r_^se^_max_/ε_β↔α_^max^*) characteristics of the studied and reference alloys [29,31,33,34,40,42,60,61,62].

Numerous studies have been set out to find the TMT conditions maximizing the functional characteristics of nickel-free Ti-based SMAs. In this context, a reversible strain, especially a superelastic recovery strain at human body temperature, represents a key metric of the TMT effectiveness. It is known that the maximum superelastic recovery strain is limited by the maximum theoretical recovery strain *ε_β↔__α_^max^*, which, in turn, depends on the alloy composition. As far as the chemical composition is concerned, the superelastic Ti–Zr–Nb-based alloys developed in recent decades can be divided into two main groups: low-Zr (≤20 at. % Zr) and high-Zr alloys (≥40 at. % Zr) with maximum theoretical recovery strains of ~6% and 7%, respectively. In Figure 12 and Table 2, the mechanical and functional properties of the Ti–Zr–Nb-based alloys provided in [29,31,33,34,40,42,60,61,62] are compared with those obtained in this work.

On the one hand, the Ti–18Zr–15Nb alloy of this study demonstrated higher superelastic strains than all other low-Zr alloys, while its mechanical resistance was somewhat lower than that of the Fe- and Sn-added low-Zr alloys [31,33,61]. On the other hand, high-Zr alloys, for example Ti–40Zr–8Nb–2Sn [29] subjected to aging, demonstrated simultaneously excellent superelasticity and high-strength characteristics. It can be hypothesized that, if the TMT conditions proposed in this work were applied to the Zr-rich alloys, it would be possible to further improve their functional characteristics. The relevance of this hypothesis can be supported by calculating the ratio of a maximum realized superelastic strain to its theoretical equivalent (*ε_r_^se^_max_*/*ε_β↔__α_^max^*). As seen in Table 2, the Ti–18Zr–15Nb alloy of this work subjected to RF700 + WQ + PDA525 reaches 71% of its maximum theoretical recovery strain of ~5.0% compared to 62% for the Ti–40Zr–8Nb–2Sn alloy [29], with the highest known maximum theoretical recovery strain of ~7.9%.

Note that this study targeted technology allowing the production of rods for spinal surgery, wherein the following characteristics are important: Young’s modulus, ultimate tensile strength, superelastic recovery strain and functional fatigue resistance. The selected TMT modes fully satisfied the first requirement, while the level of satisfaction in terms of the strength and reversible strains remained questionable. In order to identify the optimal TMT, more specifically the most appropriate rotary forging temperature, the fatigue testing of spinal implant assemblies must be conducted according to ISO 12189 (dynamic fixation) conditions, which will be carried out in a future work.

## 5. Conclusions

The results of the present study of a Ti–18Zr–15Nb (at. %) shape memory alloy (SMA) subjected to a combined thermomechanical treatment (TMT), including hot rotary forging (RF) with either air cooling (AC) or water quenching (WQ), and post-deformation annealing (PDA), led to the following conclusions:

1. With an increase in the RF temperature from 600 °C to 700 °C, the average grain size of the *β*-phase increases significantly, from 3–6 µm to 14–18 µm. After RF at 600 °C and 700 °C, a non-recrystallized substructure was formed due to dynamic polygonization. WC leads to the formation of a finer-grained microstructure compared to AC. Cold rotary forging (CRF) is characterized by the presence of strongly elongated grains in the extraction direction and small amounts of stabilized *α*″-martensite.

2. During PDA at 525 °C after high-temperature TMT, grain growth by the boundary migration mechanism is noticeable, but in general, this PDA does not affect the microstructure. The difference in the average grain size between WC and AC was preserved after PDA at 525 °C. During PDA at 750 °C, a completely recrystallized microstructure with an average grain size of 29–35 µm was formed, regardless of the previous TMT.

3. Static tensile testing showed that the alloy after TMT demonstrates a low apparent Young’s modulus (33 to 51 GPa). The specimens after CRF showed the highest *UTS* values (~800 MPa), but their stress–strain diagrams did not contain typical superelastic plateaus. The alloy after RF at 600 °C exhibits higher strength compared to its equivalent after RF at 700 °C. The transformation yield stress and dislocation yield stress are systematically higher for the AC specimens compared to their WQ equivalents. The tensile strength, dislocation and transformation yield stresses decrease after PDA at 525 °C. These results correlate with the XRD and EBSD observations, which show a significant substructural hardening of the material during RF at 600 °C and softening by polygonization processes during PDA at 525 °C. After PDA at 750 °C, all of the samples demonstrate the highest elongation to failure of 15–20% obtained at the expense of the lowest *UTS* values of 535–570 MPa, which is explained by the formation of a recrystalized coarse-grained microstructure.

4. Pronounced superelasticity is found in samples after all the TMTs except for CRF. Specimens after RF at 700 °C demonstrate larger superelastic recovery strains compared to their equivalents after RF at 600 °C. Differences in the water- and air-cooling rates do not significantly affect the superelasticity. The superelasticity of the specimens after RF at 700 °C is slightly improved by PDA at 525 °C, in contrast to their equivalents subjected to RF at 600 °C. With increasing annealing temperature to 750 °C, the difference between the dislocation and transformation yield stresses diminishes, and the superelastic recovery decreases. The best performance during cyclic tests was demonstrated after RF700 + WQ + PDA525, when the superelastic strain recovered due to reverse martensitic transformation was the highest (*ε_r_^se^_max_* = 3.4%). Such high functional properties result from the formation of a polygonized dislocation substructure of *β*-phase and a favorable <101> texture. It can be concluded that RF at 700 °C results in the best combination of the Ti–18Zr–15Nb alloy’s functional properties, regardless of the cooling rate applied.

## Figures and Tables

**Table 1:** Chemical composition of the Ti–18Zr–15Nb ingot (at. %).

Ti	Zr	Nb	C	O	H	N
Main	17.8	15.1	0.098	0.403	0.516	0.001

**Table 2:** Mechanical and functional properties of the studied and reference alloys.

T_RF_, °C	T_PDA_, °C	Cooling/CRF	*σ_tr_*, MPa	*UTS*, Mpa	*δ*, %	*ε_r_^se^_max_*, %	*ε_r_^tot^*, %	*ε_β↔α_^max^*, %	*ε_r_^se^_max_*/*ε_β↔α_^max^*, %
600	–	WQ	460 ± 9	762 ± 12	11.6 ± 3.9	2.7	6.2	4.8 ± 0.8	56
		AC	560 ± 36	747 ± 31	13.2 ± 1.5	2.8	6.4		58
	525	WQ	370 ± 41	635 ± 33	13.8 ± 1.8	2.6	6.4		54
		AC	420 ± 28	683 ± 26	16.0 ± 1.8	2.8	6		58
		CRF	530 ± 31	685 ± 25	11.1 ± 1.0	2.7	6.3		56
700	-	WQ	315 ± 49	635 ± 30	14.5 ± 1.1	3.2	6.7		67
		AC	427 ± 27	632 ± 20	14.3 ± 3.7	3.1	6.6		65
	525	WQ	315 ± 15	598 ± 32	15.6 ± 0.6	3.4	6.4		71
		AC	277 ± 15	579 ± 23	12.9 ± 6.2	3.2	6.2		67
		CRF	330 ± 18	639 ± 17	14.3 ± 2.4	2.8	6		58
**Alloy**		**Final TMT**							
Ti–18Zr–15Nb [40,60]		Cold rolling (CR) (e = 4.2; 3.0 *) + PDA 900 °C (30; 5 * min)	253 *	527 *	39 *	3.0	4.1	5.1	59
Ti–18Zr–15Nb [62]		CR (e = 0.3) + PDA 550 °C (30 min)	275	534	10.3	2.8	4,8	5.2	54
Ti–18Zr–11Nb–3Sn [31,61]		CR (e = 4.2) + PDA 800 °C (10 min)	295	670	12	1.8	2,7	~5.3	34
Ti–19Zr–10Nb–1Fe [33]		CR (e = 1.4) + PDA 600 °C (30 min)	414	703	24.8	2.7	3.7	~4.5	60
Ti–43Zr–10Nb [42]		CR (e = 0.3) + PDA 550 °C (30 min)	171	458	14.4	3.2	5	7.5	43
Ti–40Zr–8Nb–2Sn [34]		CR (e = 3.9) + PDA 900 °C (30 min)	374	835	28.0	4.2	5.5	7.9	53
Ti–40Zr–8Nb–2Sn [29]		CR (e = 3.9) + PDA 900 °C (30 min) + Aging 300 °C (60 min)	578	850	11.6	4.9	7.0	7.9	62

* TMT schedule and mechanical properties in [60].

## Data Availability

Not applicable.
